# War and Health

**Published:** 1914-05

**Authors:** 


War and Health.—The prospect for war with Mexico is of interest to the medical profession, both from the general aspect with which it is view by the government and from its effect upon public health. While naturally there will be the deepest regret at the loss of life as a direct result of the war, especially as that loss will be from among the sturdiest and healthiest young men of both countries, there is compensation in the thought that American conquest, which will inevitably follow, will result in an immediate improvement in the health conditions of Mexico.

The fact that large numbers of our American forces have been mobilized for many months in Texas, thus inuring them to the Southern climate, will prevent much loss of life from diseases generally incident to warfare in tropical countries. Our army will go into the war in the best of health, and while the loss from disease will likely be larger than from the guns of the enemy, precautions are being taken to reduce that loss to the minimum.

Unfortunately Mexico has never safeguarded the health of its people as it should have done, and as a consequence the border States have had to exercise strict quarantine regulations to prevent the spread of disease into this country. Mexico has long been regarded as a breeding place for many diseases and consequently as a menace to the health of the nation. Naturally among the first things the United States will do after the establishment of peace will be to demand such a cleaning up of Mexico as will insure better health to the people, and in a short time we may expect Mexico to be as healthy as the Panama Canal Zone and other tropical sections where the American flag dominates.

From the standpoint of public health alone the war will doubtless be worth more than it will cost in either money or life. At this time the war, if such it can really be designated, gives promise of being an almost bloodless conquest, the beneficent results of which must of necessity soon come to be apprceiated by the Latin-Americans who have sufficient enlightenment and intelligence to know a good thing when they see it. The time has come when civilized people the world over deem it a first duty to safeguard the public health, knowing that "an ounce of prevention is worth a pound of cure."

